# Clinical utility of the Arabic medication-related burden quality of life (MRB-QoL) tool in hospital-based medicines optimisation services: A mixed methods feasibility study

**DOI:** 10.1016/j.rcsop.2025.100620

**Published:** 2025-06-03

**Authors:** Sundos Q. Al-Ebrahim, Ahmad El Ouweini, Fatima Boura, Heba M. Abu Tayyem, Rami Diab, Omar Adas, Nemah Awwad, Maisam Tobeh, Fatima A.L. Salame, Sara A.L. Jabi, Ghattas Abu Dawoud, Hamzah Alzubaidi, Jeff Harrison, Timothy F. Chen, Mohammed A. Mohammed

**Affiliations:** a School of Pharmacy, Faculty of Medical and Health Sciences, The University of Auckland, Auckland, New Zealand; b College of Pharmacy, Gulf Medical University, Ajman, United Arab Emirates; c Department of Pharmacy, Thumbay University Hospital, Ajman, United Arab Emirates; d Department of Pharmacy, Sheikh Khalifa Hospital, Ajman, United Arab Emirates; e Department of Pharmacy, Al Qassimi Hospital, Emirates Health Services, Sharjah, United Arab Emirates; f Department of Pharmacy, Cleaveland Clinic, Abu Dhabi, United Arab Emirates; g Department of Pharmacy, Ibrahim Bin Hamad Obidallah Hospital, Ras Al-Khaimah, United Arab Emirates; h College of Pharmacy, University of Sharjah, Sharjah, United Arab Emirates; i School of Pharmacy, Faculty of Medicine and Health, The University of Sydney, Sydney, Australia

**Keywords:** MRB-QoL, Arabic, Clinical pharmacist, Medicines optimisation, Medication reviews, Medication related burden, Quality of life, Clinical utility, Feasibility

## Abstract

**Background:**

The Medication-Related Burden Quality of Life (MRB-QoL) Arabic version is a 31-item valid and reliable measure of medication burden on functioning and well-being in people with long-term conditions.

**Aim:**

To evaluate the feasibility of using the Arabic MRB-QoL tool in clinical pharmacist-led medicines optimisation services in United Arab Emirates (UAE) hospitals.

**Method:**

This non-randomised, non-controlled, feasibility study was conducted in 4 UAE hospitals, utilising a mixed-methods approach. The clinical utility of the MRB-QoL Arabic was evaluated, covering various aspects of feasibility, including acceptability, usability, benefits, facilitators, and barriers to its implementation in practice. The study comprised 3 stages: providing training for clinical pharmacists (CPs) and nurses, implementing the Arabic MRB-QoL tool, and the System Usability Scale (SUS) survey and semi-structured interviews with CPs. The usability and perceived benefits were evaluated using qualitative interviews and a Qualtrics survey. The perceived acceptability, barriers, and facilitators were explored through analysis of the interviews.

**Results:**

Ten CPs implemented the Arabic MRB-QoL tool during routine medication reviews for 227 admitted patients. Thematic analysis of the interview transcripts identified key themes that highlighted the acceptability, usability, benefits, as well as facilitators, and barriers the CPs faced in implementing the tool in their routine clinical practice. In addition, the SUS survey showed an average score of 82.2, indicating excellent usability of the tool in facilitating medicines optimisation services.

**Conclusions:**

This study confirmed the clinical utility of the MRB-QoL Arabic in pharmacist-led medicines optimisation services in UAE hospitals, highlighting preliminary evidence of its acceptability, usability, and benefits, as well as facilitators and barriers to implementation. By promoting patient-centred medicines optimisation, the Arabic MRB-QoL tool has the potential to help healthcare providers gain insights into patients' experiences with medicines and the key dimensions of medication burden patients encounter, optimise medicines regimens, and improve patients' quality of life.

## 1 Introduction

Medicines are essential for preventing and treating long-term conditions (LTCs). However, long-term use, especially involving complex medicine regimens, is burdensome to patients and demands continuous effort from both patients and healthcare providers to ensure medicines are used safely and appropriately. The adverse consequences of inappropriate use of medicines are well established, and they underscore the need for person-centred and collaborative care to optimise patient outcomes.^1^ Medicines optimisation is a person-centred service that incorporates patients' preferences, values, and experiences to ensure the appropriate, safe, and evidence-based use of medicines.^1^^,^^2^ With appropriate integration of patient-reported measures into decision-making, medicines optimisation services have the potential to reduce medication-related burden (MRB) and improve patient outcomes and quality of life (QoL).^2^

Some individuals with LTCs experience higher levels of MRB,^3^ which can adversely affect their social, psychological, and physical well-being.^4^, ^5^, ^6^, ^7^ With a higher burden compromising QoL, the clinical, economic, and humanistic related undesired outcomes are higher.^8^ However, many healthcare providers have limited insights into the multidimensional aspects of medication burden encountered by patients.^9^ Patient experiences are often overlooked,^10^ and psychosocial impacts of medication burden receive less emphasis than biomedical factors^11^ during therapeutic decision-making. One key approach to assessing the impact of person-centred care, such as medicines optimisation for patients' well-being, is the evaluation of changes in medicines-related QoL outcomes.^12^ However, less emphasis is often placed on assessing MRB and reducing its impact on QoL in medicines optimisation services. Recognising MRB and reducing its impact during medicines optimisation services requires valid, patient-centred measures of medication burden. Using such measures helps healthcare providers gain insights into patients' experiences with medicines and the key dimensions of MRB patients encounter, which in turn helps to optimise medicines regimens, and improve patients' QoL. Over the past two decades, there has been a growing interest in developing patient-reported measures of treatment and medication burden, particularly in English-speaking countries.^9^^,^^13^, ^14^, ^15^, ^16^, ^17^ However, the lack of comprehensive measures for use in Arabic-speaking populations calls for cross-cultural adaptation, validation, and additional psychometric evaluation of existing measures.^18^ Examples of such measures include the Medication-Related Burden Quality of Life (MRB-QoL) tool.^13^

The Arabic MRB-QoL tool is a valid and reliable patient-reported measure of medication burden on functioning and well-being.^19^^,^^20^ It was shown to be a culturally appropriate, relevant, clear, and comprehensive measure of medication burden among Arabic-speaking patients with LTCs.^19^, ^20^, ^21^ Despite its evidence of validity and reliability,^19^, ^20^, ^21^ the Arabic MRB-QoL measure's clinical utility has not been evaluated. This study aims to close this gap by evaluating the feasibility of using the Arabic MRB-QoL tool in clinical pharmacist-led medicines optimisation services in UAE hospitals. The specific objectives of the study were to:

1. Explore the acceptability, usability, and benefits of implementing the Arabic MRB-QoL in clinical pharmacist-led medicines optimisation services in UAE hospital settings.
2. Describe the barriers to and facilitators of integrating the Arabic MRB-QoL into medicines optimisation services in hospital settings in the UAE

## 2 Methods

### 2.1 Study design

This study was a non-randomised, non-controlled feasibility study conducted utilising quantitative and qualitative methods. This mixed-methods design was chosen to provide comprehensive perspectives on the topic explored and facilitate triangulation of the data.^22^ The study was reported using the CONSORT 2010 statement: extension to randomised pilot and feasibility trials.^23^

### 2.2 Participants and setting

This feasibility study was conducted in 4 tertiary hospitals across 3 cities in the UAE. The study population consisted of 3 groups: clinical pharmacists (CPs) providing medicines optimisation services, patients with LTCs admitted to these hospitals and receiving medication reviews by CPs, and nurses who were involved as data collectors.

CPs from the 4 UAE hospitals were invited to participate in this study. Eligible CPs had to be licensed in the UAE, involved in ward-based medication reviews, and willing to implement the Arabic MRB-QoL tool in their routine clinical services. CPs who did not meet these criteria were excluded.

Patients with LTCs were recruited from clinical pharmacist-led medication review services. Eligible patients had to be 18 years and older, having at least one LTC, take at least one prescription medicine regularly, be willing to participate and provide written informed consent, be able to complete the MRB-QoL in Arabic, be admitted to any of the 4 hospitals, and be enrolled in clinical pharmacist-led medication review services. Pregnant patients and patients with cognitive impairment, terminal illness, or vision or hearing impairment were not included in the study.

Data collection involved collaborations between CPs and nurses. Eligible nurses had to be employed at the study sites, have previous research experience (at least as data collectors), be willing to participate, and provide informed consent. Nurses whose duty hours conflicted with CPs' working hours were excluded.

Various methods have been used to guide sample size estimation for feasibility studies.^24^ Most of these are based on a ‘rule of thumb' with the recommended sample sizes ranging from 10 to 70.^25^ In light of the lack of previously published data specifically addressing the clinical utility of the MRB-QoL tool, in this feasibility study, we aimed to enrol 10 CPs across the 4 study locations. CPs recruited all eligible patients using a time-bound consecutive sampling approach within 5 weeks.

### 2.3 Study measures

#### 2.3.1 The Arabic version of MRB-QoL

Al-Ebrahim et al. (2024)^19^ translated and culturally adapted the original English version of the MRB-QoL^13^ for use in Arabic-speaking populations. The Arabic MRB-QoL was then content validated using a rigorous approach, which included the e-modified Delphi technique with an expert panel (9 in Round 1, 7 in Round 2) and cognitive debriefings with 5 end users. The conceptual framework of the Arabic MRB-QoL measure was supported by confirmatory factor analysis.^21^ The tool demonstrated good construct validity, including structural, known-group, convergent, and discriminant validity.^20^ It also exhibited excellent reliability, including high internal consistency, low measurement error, and good test-retest reliability. The Arabic MRB-QoL is a 31-item tool categorised into 4 domains: routine and regimen complexity (RRC, 11 items), psychosocial burden (PsySB, 9 items), functional and role limitation (FRL, 8 items), and therapeutic relationship (TR, 3 items). In this study, patient participants were asked to indicate their level of agreement with each statement of the MRB-QoL Arabic on a five-point Likert scale ranging from ‘1 = strongly agree’ to ‘5 = strongly disagree’. The resulting scores ranged from 0 to 100, with higher scores indicating a higher medication burden and a lower QoL.

#### 2.3.2 System usability scale (SUS)

In this study, CPs were asked to complete the system usability scale (SUS) survey on the usability of implementing the Arabic MRB-QoL to facilitate medicines optimisation services. The SUS is a reliable and valid tool designed to assess the perceived usability of tools, products, and services.^26^^,^^27^ The SUS comprises 10 items, rated on a 5-point Likert scale ranging from 1 to 5 (‘strongly disagree’ to ‘strongly agree’). The scale includes both positively and negatively worded items to mitigate potential biases stemming from respondents' lapses in attention during completion.^27^ The total score is computed using Brooke's standard scoring method; scores range from 0 to 100 and are grouped into 4 categories of usability: 0–50 unacceptable, 51–68 poor, 68–80.3 good, and > 80.3 excellent.^26^^,^^28^ The scores can also be converted into letter grades (from A to F) based on percentile ranks. For instance, a score higher than 80.3 is equivalent to an ‘A' grade; a score ranging from 68 to 80.3 equates to a ‘B' grade; and a score below 50 is considered an ‘F' grade, indicating poor usability of the scale. To further explore usability aspects, item-level analysis was carried out in line with established scholarly approaches.^29^^,^^30^

### 2.4 Data collection

Data collection involved coordinated teamwork between nurses and CPs. This approach was crucial to smoothly implement the Arabic MRB-QoL tool into hospital routines and to ensure its effective use in real-life settings. Nurses recommended by the study site collaborators and CPs were invited to participate in the study through email invitations. Email addresses of potential participants were obtained from the hospitals' human resources departments with the necessary permissions and approvals. Information about the study, including the expectations from pharmacists and nurses, was provided. Pharmacists and nurses willing to participate in the study were screened according to eligibility criteria, and those eligible were requested to provide written informed consent before participating. The study comprised 3 stages: Stage I involved providing training for CPs and nurses, Stage II focused on implementing the Arabic MRB-QoL tool, and Stage III included the SUS survey and semi-structured interviews with CPs (Table 1).

**Table 1:** Implementation stages for the Arabic MRB-QoL in medicines optimisation services.

Stage I	Stage II	Stage III
Ethics approval	Patients screened for eligibility by CPs	CPs completed SUS
Eligibility screening and inviting CPs and nurses	Patients approached and recruited in the study by the nurses/data collectors	Semi-structured interviews with CPs
Training CPs and nurses	Patients completed an online MRB-QoL Arabic	
	CPs received Arabic MRB-Qol scores and used them during medication reviews	

CPs; Clinical pharmacists, MRB-QoL; Medication-related burden quality of life, SUS; System use usability.

#### 2.4.1 Stage I: training

Stage I of this study involved providing training for nurses and CPs by a board-certified pharmacotherapy specialist (SQA) through an online webinar. The webinar covered information for CPs about screening patients for eligibility, notifying nurses about eligible patients, and guidance on how to use the Arabic MRB-QoL scores in the medicines optimisation services. Nurses received training on how to invite eligible patients, obtain patient consent, and facilitate access to the Arabic MRB-QoL for patients participating in the study. To ensure effective training for nurses and pharmacists in the online webinar, the following strategies were used: interactive sessions, comprehensive materials, and follow-up support.

#### 2.4.2 Stage II: implementing the Arabic MRB-QoL in medicines optimisation services

Medication reviews are part of the routine clinical services provided by CPs in all 4 hospitals included in this study. The time for CPs to provide this routine care, from admission to completion of medication reviews, varied based on hospital guidelines and patient complexity. Typically, in UAE hospitals, this service is offered within the first 24 h following admission to ensure the compilation of a comprehensive and accurate medicines list. In this study, the proposed timeline for Stage II was informed by the 24-h post-admission timeframe for medication reviews in UAE hospitals and the experience of CPs and recommendations from collaborators at each study site (Table 2).

**Table 2:** Timeline for stage II implementation of Arabic MRB-QoL in medicines optimisation services.

Timeline (24 h post-admission)	Implementation steps	Actions
0 h	Notification of new admissions	• CPs were notified via the hospital platform about newly admitted patients.
0–2 h	Screening eligibility and notifying nurses	• CPs reviewed patient information and notified the nurses (data collectors) about eligible patients.
2–5 h	Patients recruitment and Arabic MRB-QoL survey administration	• The nurse/data collectors (i) approached eligible patients and invited them to the study, (ii) provided study details to patients, and (iii) obtained patients' informed consent. • Patients completed an online Arabic MRB-QoL questionnaire, and the survey data was sent to CPs by the nurse.
5 h –(MedReviews)	Incorporating Arabic MRB-QoL data into CPs workflow	• CPs conducted MedReviews. • CPs used the Arabic MRB-QoL data to inform decision-making during medication reviews.

CPs; Clinical pharmacists, MRB-QoL; Medication-related burden quality of life, MedReviews; Medication reviews.

As part of their routine workflow, CPs are informed via the hospital platform about newly admitted patients needing medication reviews. In this study, CPs accessed patients' clinical and medicines information via the hospital portal system to check their eligibility. Then the nurses involved as data collectors for the study approached eligible patients after being identified by the CPs. The nurses invited patients to participate, provided study details, and facilitated access to the Arabic MRB-QoL tool. The Arabic MRB-QoL survey was administered online via Qualtrics using tablets provided to eligible patients at each study site. Patients completed the survey independently without the assistance of the data collectors and/or their caregivers. To minimise any potential bias in participant responses, the data collectors were directed to provide clear instructions, maintain privacy, and limit their assistance to technical support only. After completing the Arabic MRB-QoL survey, the data on the overall MRB-QoL score and its domains were sent to the CPs and a member of the research team (SQA) (S2 supplementary data). CPs received the Arabic MRB-QoL results immediately after the patient completed the tool, allowing them to incorporate the findings into the routine medication reviews. Following this, CPs completed a data collection template for each patient. This template included socio-demographic information, types and numbers of LTCs, numbers and types of medicines, causes and types of drug-related problems (DRPs), and recommendations. The data collection template included a pre-coded list of DRP types, causes and interventions based on the Pharmaceutical Care Network of Europe (PCNE) classification system.^31^ CPs were responsible for selecting the most relevant options during the medication review process. It also contained responses to closed-ended questions about the tool's benefits during medication reviews and open-ended questions on how the overall score and the 4 domain scores benefited their practice.

#### 2.4.3 Stage 3: semi-structured interviews and SUS survey with CPs

CPs were asked to complete the SUS survey to assess the usability of implementing the Arabic MRB-QoL tool in medicines optimisation services. CPs who completed the survey were invited to participate in a one-on-one semi-structured interview with a member of the research team (SQA) to investigate the acceptability, usability, benefits, barriers, and facilitators of implementing the Arabic MRB-QoL tool in routine medicines optimisation services. Custom-designed semi-structured interview questions were developed based on the literature and the research team's experience in the area (Table S1 supplementary data). The interview guide was evaluated for face validity and pilot-tested with 2 registered pharmacists who were not included in the study. Interviews were recorded using a digital recorder and transcribed. Each CP was assigned a unique identifier number, and a document linking each number to the CP's corresponding interview recordings and transcriptions was created.

### 2.5 Data analysis

The acceptability, barriers, and facilitators of integrating the Arabic MRB-QoL tool into routine practice were explored through thematic analysis of the interview transcript. The usability and perceived benefits of the tool among CPs were evaluated using both interviews data and survey responses. The survey responses included SUS to assess usability and additional open-ended and closed-ended questions to assess participants' perceptions of the benefits of the MRB-QoL for medicines optimisation services. In the closed-ended questions, CPs were asked whether they found the MRB-QoL beneficial during medication reviews. The open-ended questions invited CPs to share their experiences of the benefits gained by integrating the MRB-QoL tool into their practice.

**Fig. 1:**
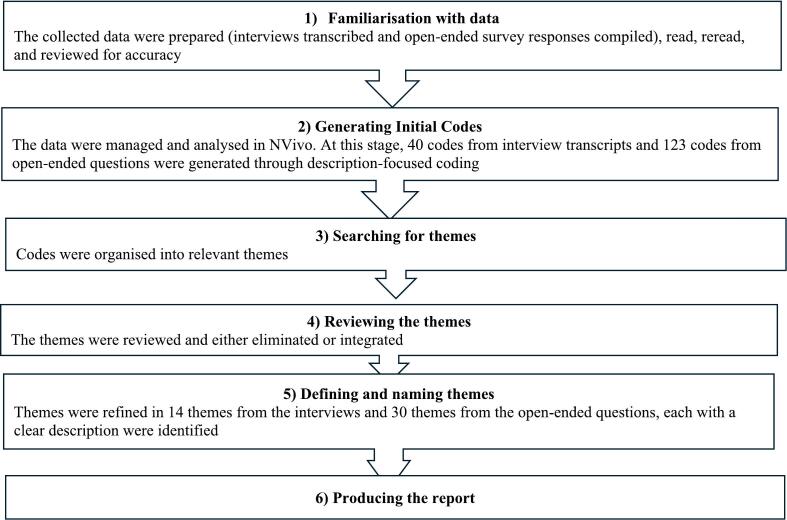
Thematic analysis steps (Braun and Clarke, 2006).^32^

Thematic analysis was conducted following the 6 phases of the method proposed by Braun and Clarke.^32^ This analysis included familiarisation with the data, generating initial codes, identifying themes, reviewing themes, defining and naming themes, and generating the final report (Fig. 1). A member of the research team (SQA) conducted the initial analysis, and other co-authors (MAM, TFC, and JH) reviewed each phase with SQA to finalise the emerging codes and themes. The development of themes was achieved through a collaborative and iterative process, with authors (SQA, MAM, TFC, and JH) meeting regularly to discuss findings, validate interpretations, and reach consensus on the final outcomes. The use of field notes recorded during data collection, along with ongoing collaborative discussions among the research team members, encouraged reflexivity and confirmed the development of codes.

A stem and leaf plot^33^ was constructed to visualise the frequency of the codes, instead of relying solely on direct counts.^34^^,^^35^ This ensured that the generation of themes was not disproportionally influenced by infrequently occurring codes, thus promoting rigorous discussion and facilitating appropriate adjustments to the coding process.^34^

The collected data were transferred from the data collection sheets to data management and analysis software. Descriptive statistics were used to summarise the SUS survey responses and analysis of close-ended questions. The data were analysed using the IBM® Statistical Package for Social Sciences Statistics (SPSS® Version 29, Armonk, NY, USA). Thematic analysis was conducted using NVivo software version 14.

### 2.6 Ethics approval

Ethics approval was obtained from the Auckland Health Research Ethics Committee at the University of Auckland, New Zealand (Approval No. AH26533), the Research Ethics Committee of the UAE Ministry of Health and Prevention (Approval No. MOHAP/DXB-REC/O.N·D/No.101/2022), and the Dubai Scientific Research Ethics Committee at the Dubai Health Authority, UAE (Approval No. DSREC-01/2023_13).

## 3 Results

### 3.1 Participant characteristics

Table 3 presents the demographic of the CPs and the clinical characteristics of the patients involved in the study. Ten CPs implemented the Arabic MRB-QoL tool during routine medication reviews of admitted patients (*N* = 227) across the 4 hospitals, with the number of patients recruited by each pharmacist ranging from 14 to 27. The average age of CPs was 33 years, and there was a median of 5 years (IQR 0.6–9.4) of experience in practice. Women comprised a slightly larger proportion of the sample (60 %). The median (IQR) age of participating patients was 45 (34–58) years, and over one-third (34 %) of the patients were UAE citizens. The employment status of patients varied, with 45.8 % being employed. The median number (IQR) of medicines and medical conditions were 5 (3–6) and 3.4 (2–4), respectively. Forty-eight percent of the patients were on 5 or more medicines (*n* = 109), and 68.7 % had 3 or more LTCs. About half of the patients (49.3 %) had DRPs related to treatment effectiveness, followed by experiencing adverse reactions (33.9 %). Drug selection errors (50.3 %) were the leading cause of DRPs, followed by dose errors (30.34 %) (Table S3 supplementary data).

**Table 3:** Characteristics of study participants.

Characteristics	Value
Clinical Pharmacists (N = 10)	
Age in years, median (IQR)	33 (28.1–37.9)
Female sex, n (%)	6 (60 %)
Years of work experience, median (IQR)	5 (0.6–9.4)
Years since graduation, median (IQR)	9 (4.6–13.4)
Patients (N = 227)	
Age in years, median (IQR)	45 (34–58)
Female sex, n (%)	103 (45.4 %)
**Ethnicity**	
Arab (UAE), n (%)	79 (34.8 %)
Arab (non-UAE), n (%)	141 (62.1 %)
Asian, n (%)	3 (1.3 %)
Others, n (%)	4 (1.8 %)
**Occupation**	
Unemployed, n (%)	93 (41 %)
Employed, n (%)	104 (45.8 %)
Retired, n (%)	16 (7 %)
Student, n (%)	42 (0.9 %)
Presence of co-morbidities, n (%)	219 (96.5 %)
≥ 3 medical conditions, n (%)	156 (68.7 %)
Number of medical conditions, median (IQR)	3.4 (2–4)
Number of medicines, median (IQR)	5 (3–6)
Polypharmacy (≥ 5 medicines), n (%)	109 (48 %)
**Types of DRPs**⁎	
Treatment effectiveness, n (%)	112 (49.3 %)
Adverse reactions, n (%)	77 (33.9 %)
Treatment cost, n (%)	73 (32.2 %)
Other, n (%)	100 (44 %)
**Causes of DRPs**⁎	
Drug selection^a^ n (%)	114 (50.3 %)
Dosing error^b^ n (%)	69 (30.34 %)
Drug use process^c^ n (%)	46 (20.2 %)
Logistics^d^ n (%)	27 (11.9 %)

a Drug selection issues include inappropriate drug choice, no indication for drug, wrong drug combination, drug duplication, multiple drug use, and cost-effectiveness concerns.

b Dosing errors include inappropriate drug form, low dose, wrong frequency, lack of monitoring, pharmacokinetics dose adjustment, and disease-specific dose adjustment.

c Drug use process issues include inappropriate dosing interval, drug underuse, drug overuse, patient unable to take the prescribed dosage form, and wrong drug taken.

d Logistics-related issues include medication unavailability and prescribing errors due to missing necessary information.

⁎ Percentages for types and causes of DRPs do not add up to 100 % because patient participants could have more than one drug-related problem (DRP).

### 3.2 Qualitative findings: interview scripts and open-ended responses on perceived benefit

Thematic analysis of the semi-structured interview transcripts identified 14 themes directly related to the following study's objectives: acceptability, usability, perceived benefits, facilitators, and barriers (Table 4 & Fig. S1 supplementary data**)**. Furthermore, an analysis of the open-ended question responses, which were aimed at assessing participants' perceived benefits of the MRB-QoL for medicines optimisation services, identified 30 themes. These themes were mapped to the overall MRB-QoL and its 4 domains, highlighting the significance of MRB-QoL in supporting CPs to conduct comprehensive medication reviews (Table 5 & Table S4 supplementary data). 3.2.1, 3.2.2, 3.2.3, 3.2.4, 3.2.5 describe the major themes and codes mapped to the research objectives. Composite quotations—statements synthesised from multiple participants—were selected to support these themes, as they highlight key concepts, ensure confidentiality, and provide a cohesive narrative.^36^^,^^37^ While these quotations were deidentified, Table 4 presents ‘participant mentions of key themes,’ where numbers indicate participant codes linked to key themes. Each code corresponds to a participant who mentioned the theme, showing its occurrence and distribution in responses.

**Table 4:** Summary of identified codes and themes mapped to study objectives.

Objectives	Themes	Code	Participant mentions of key themes⁎
Acceptability	Positive perception and practical value	Enthusiasm for using	102,223,455,589
		Welcomed by pharmacists	2,222,344,444,555,566,777,788,888,999
	Versatility and contextual fit	Adaptable across settings	222,222,233,444,444,555,555,566,777,788,888
		Suggest other language versions	222
Usability	Confidence in effectiveness and clinical value	Confidence with use	8 4
		Valuable	4,455,667,889
	Ease of use and simplicity	Clear	11,357
		Easy to use	133,355,578
		Simple	234,457
	Workflow integration and efficiency	Integration with workflow	48
		Not time consuming	3,344,478
Perceived Benefits	Enhances patient outcomes and care quality	Better communication	10 1,088,888
		Improves patient outcomes	1,025,555,566,888
		Improves healthcare services	8
		Improves medicines optimisation	107,788,888
	Facilitates clinical decision-making	Identifies critical issues	2,444,455,568,899
		Identifies gaps in treatment plans	67,899
		Improves decision-making	22,344,445,889
		Informs therapeutic adjustments	102,244,556,888
	Provides holistic insights into patient-centered care	Provides comprehensive patient insights	10,335,555,668
		Focuses on key patient burdens	103,334,445,779
		Focused patient counselling	358
		Enables incorporation of patient's preferences	68
	Streamlines efficiency and productivity	Avoids irrelevant tasks	4588
		Improves productivity	48
		Simplifies the process	10 48
		Time-saving optimisation	10,888
Facilitators	Cultural relevance and patient acceptance	Culturally appropriate	105,558,889
		Patient acceptance	15,788
	Promotion of collaboration	Team collaboration involvement	34,578
		Multidisciplinary use of the tool	34
	Ease of integration with hospital workflow	Streamlined workflow integration	24,455,567
		Efficiency integration	3
	User-friendly design and simplicity	Multiple format options	1
		Simple, clear questions	12,223,334,455,789
		User-friendly design	5557
		Well-structured tool	10 6 8
Barriers	Challenges in score interpretation	Lack of scoring benchmarks	1,178,999
		Lack of detailed score breakdown	34,456,679
		Lack of ‘Not applicable’ option	27

⁎ The numbers represent participant codes linked to key themes. Each code corresponds to a participant who mentioned the theme, indicating both its occurrence and distribution in the responses.

**Table 5:** Key themes illustrating how the scores of MRB-QoL and its domains facilitate clinical pharmacists' medication reviews and optimisation for the patients (*N* = 227).

RRC	FRL	PsySB	TR	MRB-QoL
Regimen simplification	Side effect management	Counselling and support	Building trust and empathy	Managing side effects
Adherence support	Condition and therapy review	Health education	Holistic health approach	Enhancing adherence
Clear usage instructions	Medicines and health education	Polypharmacy management	Patient-centred communication	Improving quality of life
Medicines necessity assessment	Specialist referral and support	Addressing stigma	Positive health reinforcement	Patient-centred communication
Dosage form adjustments	Functional improvement support	Chronic condition support	Provider engagement support	Deprescribing
Lifestyle integration support	Lifestyle and physical health monitoring	Family and community engagement	Shared decision-making	Chronic condition support

#### 3.2.1 Acceptability

Two core themes were identified that reflect pharmacists' acceptability of the Arabic MRB-QoL tool.

##### 3.2.1.1 Theme 1: positive perception and practical value

Nine out of the ten CPs welcomed the utilisation of the tool during medication reviews, and/or they indicated their enthusiasm and willingness to use the tool beyond this study and appreciated its practical value.

‘I would continue to provide this service after the study. I highly recommend this tool.’

##### 3.2.1.2 Theme 2: versatility and contextual fit

The pharmacists (*n* = 7) regarded the tool as resourceful across healthcare settings beyond the current setting within which they applied it, and they indicated their acceptance of its integration into their workflow. For example, a participant highlighted the potential applicability of this tool across various specialties within hospitals as well as in outpatient and community settings.

‘I think it will be welcomed by many clinical pharmacists because they can use it on all floors. Whether in an intensive care unit, general ward, or maternity ward in any healthcare setting, they can even use it in the community and outpatient settings. I think if we have later other versions for non-Arab patients, it will definitely be something very useful and welcomed by the team here.’

#### 3.2.2 Usability

The usability of the Arabic MRB-QoL tool among CPs was assessed through the thematic analysis of interviews (3.2.2.1, 3.2.2.2, 3.2.2.3) and the quantitative analysis of the SUS responses (Section 3.3). The thematic analysis provided deeper insights into pharmacists' usability evaluation, with 3 major themes emerging.

##### 3.2.2.1 Theme 1: confidence in effectiveness and clinical value

Six out of ten CPs considered the Arabic MRB-QoL as valuable for informing decisions about medicines, thus indicating their confidence in its therapeutic relevance.

‘I relied on the data obtained from the MRB-QoL report when making decisions. It's a valuable tool’.

##### 3.2.2.2 Theme 2: ease of use and simplicity

Seven out of the ten CPs described the tool's usability as clear, user-friendly, and/or simple*.*

‘I really appreciate the fact that the sentences are straightforward and easy to follow; patients will have no doubt what it means. That was easy to use, easy to apply, with no need for special training, and straight forward questions’.

##### 3.2.2.3 Theme 3: workflow integration and efficiency

The Arabic MRB-QoL tool was described by 4 CPs as being uncomplicated and/or time-efficient to integrate into the hospital workflow.

‘Integrating this tool with hospital workflow was uncomplicated and of great value. It went very smoothly, and it would not be a time burden for us’.

#### 3.2.3 Perceived benefits

The perceived benefits of the tool were evaluated using data collected from qualitative interviews, as well as open-ended (3.2.3.1, 3.2.3.2, 3.2.3.3, 3.2.3.4) and closed-ended questions within the data collection template (Section 3.3). This section highlights the key qualitative findings about the perceived benefits of the Arabic MRB-QoL from interviews and open-ended responses. The thematic analysis of the interview transcripts identified the following 4 themes:

##### 3.2.3.1 Theme 1: enhanced patient outcomes and care quality

Enhancing patient outcomes and care quality was recognised by 6 CPs as a benefit of integrating the Arabic MRB-QoL into their clinical practice. The CPs found the Arabic MRB-QoL to be a valuable tool for improving their communication with patients during medicines optimisation services. They also considered it essential for enhancing healthcare service quality, patient outcomes, and overall patient communication.

‘It is an optimal solution for improving the level of healthcare services, increasing patient outcomes, improving patient communication, improving medication optimisation’.

##### 3.2.3.2 Theme 2: facilitated clinical decision-making

Nine out of the ten CPs acknowledged that facilitating clinical decision-making was a benefit of integrating the Arabic MRB-QoL tool into their daily clinical practice. Emphasised the importance of the MRB-QoL in identifying the challenges the patients have with treatment and care plans, medicines needing adjustments, and facilitating informed decision-making.

‘I believe it's a very good and useful tool that helps me identify the main and critical problems that the patient has in their medication regimen. It helped me identify which medications might need adjustments. Has informed my decision-making by highlighting patient-specific medication burden, directly influencing personalised medication recommendations. It can make my decision a proper decision for their drug-related problems.’

##### 3.2.3.3 Theme 3: provided holistic insights into patient-centred care

The implementation of the Arabic MRB-QoL was perceived by 8 CPs as beneficial for providing holistic insights into patient-centred care. CPs reported the usefulness of the MRB-QoL because it offered comprehensive insights into patients' perspectives, aided understanding of patients' experiences and preferences, and addressed the types of MRB patients encounter and their counselling needs.

‘It affords a comprehensive insight into patients’ emotional and physical load of medication and other related risks they are bound to encounter. I will focus on the highest points, mostly the patients having issues with that domain of high score. I don't need to discuss the side effects of the medication while the patient is not facing this issue. I will focus on what exactly makes patients' lives more difficult by using such medication. Instead of giving just general counselling or general advice for the patient, who may not need it, the tool makes pharmacists' counselling focused.’

##### 3.2.3.4 Theme 4: streamlined efficiency and productivity

Four pharmacists found the Arabic MRB-QoL useful for simplifying and streamlining the medicines optimisation process while also eliminating unnecessary tasks and enhancing productivity.

‘The MRB-QoL Arabic version helps reduce the time needed for medication optimisation by quickly identifying key patient concerns and burdens, leading to more focused and efficient interventions. It improves clinical pharmacists’ tasks, such as reviewing services and reducing nonrelevant tasks by sticking to patients' issues derived from the tool. It made the process very easy ‘.

The qualitative findings of perceived benefits were derived from thematic analysis of interviews and open-ended responses. Key themes identified through thematic analysis of the open-ended questions about perceived benefits were mapped to the overall MRB-QoL and its domains, and this provided insight into the pharmacists' experiences using the Arabic MRB-QoL in medicines optimisation services. Table 5 outlines how the scores of Arabic MRB-QoL and its domains facilitated clinical pharmacists' medication reviews and optimisation provided to the patients (*N* = 227). The identified themes highlight how this tool may aid medication reviews and patient-centred care. For instance, pharmacists highlighted that the ‘Routine and Regimen Complexity’ (RRC) domain of the MRB-QoL helped them to simplify regimens, provide clearer instructions on usage, make necessary adjustments regarding dosage form, counselling adherence support, and lifestyle and monitoring needed. The pharmacists also found the ‘Therapeutic Relationship’ (TR) domain helpful for building true empathy, patient-centred communication, and shared decision-making.

#### 3.2.4 Facilitators

Four key themes emerged as the facilitators of the successful implementation of the MRB-QoL tool in medicines optimisation services by CPs*.*

##### 3.2.4.1 Theme 1: cultural relevance and patient acceptance

Six CPs identified cultural relevance and patient acceptance as key facilitators for the successful integration of the Arabic MRB-QoL in medication review services. These CPs reported that the cultural relevance of the Arabic MRB-QoL, along with patient acceptance, served as key enablers for improving the tool's usability.

‘The fact that this questionnaire was culturally tailored for Arabs has an important role in actually being accepted by the patients and facilitated using the tool. What made the process easy to implement is that patients were willing and accepting of the use of the tool.’

##### 3.2.4.2 Theme 2: promote collaboration

Five CPs highlighted that collaboration between CPs and other healthcare providers, along with the multidisciplinary application of the tool, supported its effective implementation.

‘Pharmacists and nurses’ collaboration supported the medication burden assessment service efficiently. Being applicable to use this tool in all hospital settings:outpatient clinics, emergency, and inpatient will enhance its clinical implication’.

##### 3.2.4.3 Theme 3: ease of integration with hospital workflow

Six CPs highlighted that the Arabic MRB-QoL tool was effectively integrated into routine hospital workflows and/or was smoothly implemented within pharmacist-led medicines optimisation services.

‘Integrating this tool with hospital workflow was going smoothly and of great value. If we implement this approach in healthcare providers’ daily routines while assessing patients it won't be distracting our routine work, I think it will go smoothly as long as we choose the appropriate time to gather information from patients.’

##### 3.2.4.4 Theme 4: user-friendly design and simplicity

All participating CPs found the clarity of the tool, as well as the simplicity of its breakdown into domain-level and overall scores, and/or its user-friendly and well-structured design, to be key facilitators for its implementation in medicines optimisation services.

‘The tool itself facilitated the experience. They are short questions, easy to understand, and straightforward to follow. Having a hard copy and online format made its use easy’.

#### 3.2.5 Barriers

The key challenges faced by the CPs in implementing the MRB-QoL tool were summarised under the following theme.

##### 3.2.5.1 Theme 1: challenges in score interpretation

Nine pharmacists identified some obstacles in implementing the tool during medication reviews, particularly with regard to score interpretation. These challenges emerged from the absence of scoring cut-offs to indicate ‘Low’, ‘Moderate’ or ‘High’ burden and the lack a ‘not applicable’ response option in the Arabic version to give participants the option to skip questions they prefer not to answer.

‘Having no detailed customised explanation of scores output was challenging. Displaying the scores with a detailed summary of what items under the domain are critically rated negatively will save pharmacists time in avoiding manually reviewing and screening patients’ responses for each domain. Having clear cutoff point guidance would be of great help in interpreting scores effectively. Adding a “not applicable” option could serve to improve the tool quality’.

### 3.3 Quantitative findings: SUS survey and closed-ended responses on perceived benefits

This section presents the key quantitative findings regarding the usability and perceived benefits of the MRB-QoL, based on SUS survey responses and closed-ended data from collection template responses, respectively.

**Fig. 2:**
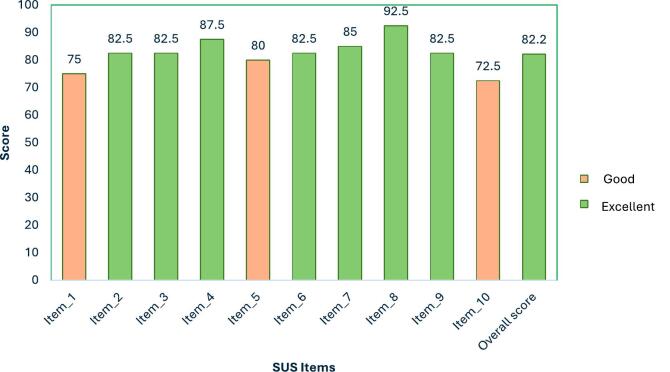
System Usability Scale (SUS) scores. SUS scores shown represent the average scores across the 10 clinical pharmacist participants. SUS scores range from 0 to 100.

Fig. 2 represents the SUS scores of participating CPs. Average scores for CPs of the individual items ranged from 72.5 to 92.5, with an average overall score of 82.2, categorising it within the excellent range (Grade A) and signifying the tool's high usability level among this group.

**Fig. 3:**
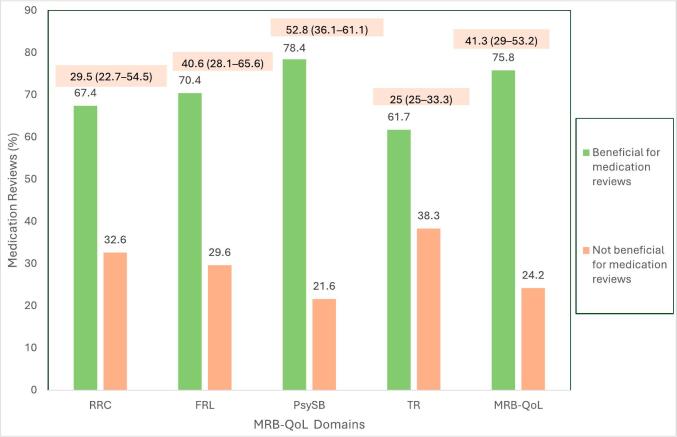
The overall and domain level Arabic MRB-QoL scores and their perceived benefits in medication reviews. Median (interquartile range) MRB-QoL scores are shown in the peach boxes above each domain. Green bars represent the percentage of medication reviews where the scores were perceived as beneficial for clinical decision-making; orange bars represent reviews where they were not. MRB-QoL = Medication-Related Burden Quality of Life; RRC = Routine and Role Complexity; FRL = Functional and Role Limitation; PsySB = Psychosocial Burden; TR = Therapeutic Relationship.

Fig. 3 provides an overview of the scores of Arabic MRB-QoL and its domains, along with the assessment of the perceived benefits of these scores for medication reviews. The median (IQR) MRB-QoL score was 41.3 (29–53.2), with the PsySB domain having the highest median (IQR) score of 52.8 (36.1–61.1) and the TR domain the lowest 25 (25–33.3). The proportion of medication reviews where MRB-QoL scores were deemed beneficial varied across the 4 domains, with the TR domain considered beneficial in 61.7 % of the medication reviews and PsySB domain considered beneficial in 78.4 % of the medication reviews. Overall, MRB-QoL scores were found beneficial in 75.8 % of the medication reviews for gaining insights into patient perspectives and making informed decisions during the medication review process.

## 4 Discussion

Patient self-reported data on MRB provide valuable insights that can inform patient-centred decision-making about medicines; however, such data are not routinely collected in clinical practice. The Arabic MRB-QoL is a valid and reliable measure of MRB on functioning and well-being designed for use in Arabic-speaking patients with LTCs. The tool encompasses multiple dimensions of MRB and provides comprehensive insights into the challenges and consequences of medication burden that patients with LTCs encounter. The current study was conducted across 4 hospitals in the UAE to assess the feasibility of incorporating the Arabic MRB-QoL tool into hospital-based medicines optimisation services. A mixed-methods design was used, including qualitative interviews and a survey of CPs. Thematic analysis of the interview transcripts identified key themes that highlighted the acceptability, usability, and benefits of the tool, as well as the facilitators and barriers the CPs faced in implementing the tool in their routine clinical practice. In addition, the SUS survey showed an average score of 82.2, indicating excellent usability of the tool in facilitating medicines optimisation services.

Integrating patient-reported outcome measures (PROMs) into clinical practice is challenging. Despite research demonstrating their benefits to improve patient outcomes,^38^^,^^39^ PROMs are often not successfully incorporated into routine clinical practice.^40^ Successful translation of evidence-based research into practice necessitates a thorough assessment of the factors affecting the implementation, including the barriers and facilitators. The UK Medical Research Council highlights the importance of conducting early feasibility assessments to improve the likelihood of successful implementation.^41^ In implementation science, feasibility studies often focus on evaluating the process rather than the intervention itself to provide valuable insights into factors that affect implementation. This enables researchers to observe processes, identify barriers, develop strategies to streamline implementation, and ultimately save resources, such as time, money, and human effort.^42^ This study is the first to assess the feasibility of implementing the Arabic MRB-QoL tool in clinical pharmacist-led medicines optimisation services within UAE hospitals, providing insights into its clinical utility. Currently, no Arabic measures of medicines and treatment burden have included data on the clinical utility of the measures.^43^, ^44^, ^45^, ^46^ Undertaking a feasibility assessment of such measures is important to increase the likelihood of their successful implementation in clinical practice.

When assessing the clinical utility of a new measure, determining the measure's acceptability and practitioners' willingness to adopt it is essential for its successful implementation in clinical practice.^47^ Our study findings revealed a high acceptability of the Arabic MRB-QoL among CPs who used it in hospital-based medicines optimisation services. In this study, the pharmacists' perceived acceptability of the Arabic MRB-QoL was explored through qualitative interviews. Pharmacists' positive perceptions about the MRB-QoL and their willingness to use it, as well as their views about the potential broader application of the tool beyond the study context, are some of the key themes identified from the interviews. Previous research on the acceptability of patient-reported measures has used various indicators, such as overall satisfaction, willingness to use, and perceived usefulness.^48^^,^^49^ Compared to studies using quantitative approaches, our method provided detailed insights into participants' experiences by encouraging in-depth discussions and enabling evidence to emerge naturally in a comfortable and open environment.^50^

Research indicates that healthcare providers' acceptance of patient-reported measures is influenced by their familiarity with the measure and its intended use in clinical decision-making.^51^ For effective application in the real world, such measures must be user-friendly, feasible to implement, and capable of accurately capturing concerns relevant to the target population.^52^ Our previous research demonstrated the cultural relevance, clarity, comprehensiveness, validity, and reliability of the Arabic MRB-QoL as a measure of medication burden among Arabic-speaking patients with LTCs.^19^^,^^20^ In this study, pharmacists successfully integrated the Arabic MRB-QoL into their daily clinical practice and reported that the tool is user-friendly and useful in facilitating patient-pharmacist communication and informing decision-making about medicines. A high overall score across SUS measures and positive feedback from pharmacists provided strong evidence of the usability of the tool. The qualitative data showed pharmacists' confidence in the clinical value of the tool. This finding also resonates with the survey results, indicating a high proportion (75.8 %) of conducted medication reviews benefited from the data of the Arabic MRB-QoL tool as it provided insights into patient perspectives and informed decision-making during medication reviews.

Pharmacists reported that the information gained from the Arabic MRB-QoL facilitated clinical decision-making and provided insights into patient-perspectives about medication burden. The tool was deemed a valuable means of both identifying significant medicines-related concerns that might otherwise have been overlooked and enhancing communication between patients and pharmacists. The MRB-QoL facilitated tailored medicines optimisation services and supported pharmacists in addressing patient needs and setting treatment goals based on these needs to enhance patient outcomes and QoL. The pharmacists also reported that the tool was useful for facilitating patient-pharmacist communication and patient involvement in shared decision-making; understanding patient perspectives; providing support to patients about adherence to treatment plans; and monitoring the adverse outcomes of medication burden These added values of the tool might empower healthcare providers with information regarding patients' lived experiences with medicines for proactive screening of at-risk individuals and for prioritising patient referrals based on the intensity of medication burden, enhancing the efficiency of care pathways for medicines review. Previous studies have shown that the integration of patient-reported measures into clinical practice improves communication between patients and healthcare providers,^53^, ^54^, ^55^, ^56^ facilitates shared decision-making,^57^ and enhances patient well-being.^56^^,^^58^ Future research could assess the impact of providing MRB-QoL scores to pharmacists through an RCT, to evaluate whether this improves DRP identification and patient outcomes.

Despite strong evidence of the acceptability, usability, and usefulness of the Arabic MRB-QoL, its integration into routine clinical practice was not without challenges, particularly with regard to the interpretation of the scores. Pharmacists reported that the lack of data on the score cut-offs to guide the prioritisation of patients based on their score category would be one potential obstacle to implementation. This needs further investigation in future studies that explore the clinical utility of the MRB-QoL. Providing data to guide the categorisation of the MRB-QoL scores and their interpretability is crucial to enhancing the evidence-based use of the tool and its utility for practice and research. Future work should also include longitudinal studies to determine the minimal clinically important difference (MCID) and provide more robust data on the minimal detectable change (MDC).

### 4.1 Implications for clinical practice and research

This study implemented a novel Arabic MRB-QoL measure into clinical pharmacist-led medicines optimisation services in UAE hospitals and presented evidence of its clinical utility and the feasibility of its integration into practice. This tool enables researchers and healthcare providers to assess MRB, evaluate the impact of medicines-focused interventions on QoL, optimise medicines regimens, and improve the well-being of Arabic-speaking patients. The findings of this study set the groundwork for future research into the integration of the Arabic MRB-QoL tool into practice to facilitate medicines optimisation services and improve patient outcomes in the UAE and other Arabic-speaking countries. The Arabic MRB-QoL can be used as an outcome measure for evaluating medicines optimisation services and screening patients at high risk of MRB. In clinical trials, the Arabic MRB-QoL can also serve as a complementary measure alongside validated objective measures of medication burden, such as the Drug Burden Index^59^ and the Medication Regimen Complexity Index.^60^ Further research should also explore the long-term benefits of integrating the MRB-QoL into clinical practice, with a particular focus on its added value in optimising medicines use and improving patient outcomes. In addition, applying the tool across diverse patient populations and healthcare systems may help identify and mitigate the clinical and patient-related factors that contribute to medication burden and its impact on QoL.

### 4.2 Strengths and limitations

This study provided preliminary evidence of the clinical utility of the Arabic MRB-QoL by covering various aspects of feasibility, including its acceptability, usability, and benefits, as well as the facilitators of and barriers to its implementation in practice. A robust mixed-method approach was used to provide comprehensive insights into the tool's clinical utility as well as the feasibility of integrating it into practice. The study generated preliminary data to inform future research needed to improve the performance and usability of this tool. Pharmacists' endorsement of the tool, as shown in both the qualitative and quantitative data, highlights the value of its role in providing healthcare providers with insights into patient perspectives and encouraging patients to participate in shared decision-making about their medicines.

Despite these strengths, our study also has several limitations. The number of pharmacists involved in the implementation of the tool was relatively small. However, this may have had no impact, as each pharmacist implementing the tool recruited a sufficient number of patients across 4 hospitals used as our study sites. Additionally, as feasibility studies primarily aim to identify potential challenges in the implementation process, they do not require the estimation of statistical power as is typically necessary in other types of studies.^61^^,^^62^ Only the interviewer involved in conducting all interviews analysed the data. However, potential bias was mitigated by adhering to an a priori interview guide and analysis framework.^63^ This study utilised data from hospital inpatients, whereas the original MRB-QoL tool was developed using data from community-dwelling individuals, and prior Arabic validation studies have focused on outpatient populations. While this distinction broadens the potential applicability of the tool, it is important to acknowledge that MRB may vary across different patient groups. Future validation studies should explore how such variations—across populations and healthcare settings—may influence the tool's performance, validity, and relevance. A further limitation of this study is the absence of a formal framework to guide MRB-QoL tool implementation.

## 5 Conclusion

This study has demonstrated the feasibility of integrating the Arabic MRB-QoL into routine hospital-based medicines optimisation services by highlighting evidence of its acceptability, usability, and benefits, as well as the facilitators of and barriers to its implementation. By promoting patient-centred medicines optimisation, the Arabic MRB-QoL tool has the potential to enhance communication between patients and healthcare providers and to facilitate shared decision-making about medicines. Future research should explore strategies to overcome barriers to implementation and optimise the clinical utility of this tool.

## CRediT authorship contribution statement

**Sundos Q. Al-Ebrahim:** Writing – review & editing, Writing – original draft, Project administration, Methodology, Investigation, Formal analysis, Data curation, Conceptualization. **Ahmad El Ouweini:** Project administration. **Fatima Boura:** Project administration. **Heba M. Abu Tayyem:** Project administration. **Rami Diab:** Project administration. **Omar Adas:** Project administration. **Nemah Awwad:** Project administration. **Maisam Tobeh:** Project administration. **Fatima A.L. Salame:** Project administration. **Sara A.L. Jabi:** Project administration. **Ghattas Abu Dawoud:** Project administration. **Hamzah Alzubaidi:** Supervision. **Jeff Harrison:** Supervision. **Timothy F. Chen:** Supervision. **Mohammed A. Mohammed:** Writing – review & editing, Visualization, Supervision, Methodology, Conceptualization.

## Declaration of competing interest

The authors declare that they have no known competing financial interests or personal relationships that could have appeared to influence the work reported in this paper.

## Appendix A Supplementary data

Supplementary material Image 1
