# Supplementary material for: Clinical utility of the Arabic medication-related burden quality of life (MRB-QoL) tool in hospital-based medicines optimisation services: A mixed methods feasibility study
Source: Explor Res Clin Soc Pharm. 2025 Jun 3;19:100620. doi: 10.1016/j.rcsop.2025.100620 (PMC12205538; doi:10.1016/j.rcsop.2025.100620)
Supplement: Supplementary file 1 — Supplementary material [file mmc1.docx]

**Appendix A. Supplementary Data**

**Table S1**

Semi-structured interview questions

| Perceived benefits | - To what extent do you believe the MRB-QoL Arabic version enhances your ability to optimise patient medicines? - How valuable is the MRB-QoL Arabic version in improving patient care within your clinical pharmacist-led services? |
| --- | --- |
| Perceived impact on clinical decision-making | - Did you use the information on the Arabic MRB-QoL report for decision making? - Has the MRB-QoL Arabic version influenced your medicines recommendations for patients? - In your opinion, does the MRB-QoL Arabic version provide valuable insights into medication-related burdens? |
| Task efficiency and time savings | - Do you think using the MRB-QoL Arabic version streamlines your medicines optimisation tasks? - Has the MRB-QoL Arabic version reduced the time required for medicines optimisation services? |
| Barriers to medication-related burden assessment in hospital | - What barriers or challenges did you encounter while conducting the MRB assessment services? - How could these barriers be overcome in the future? |
| Facilitators to the uptake of the use of Arabic MRB-Qol tool in hospitals | - What facilitators do you think supported this service? And why? |
| The CPs' role in screening MRB and the use of Arabic MRB-Qol tool | - Is this a service you would continue to provide in your hospital after this study? And how could it be improved? |

**S2**

Mock-up of MRB-QoL Arabic Summary Report Provided to Clinical Pharmacists

Dear Dr XXXX,

A new patient survey has been completed. Here are the scores:

Patient Number:12166

Routine and regimen complexity (REC) score: 70.45454545454545454545

Functional and role limitation (FRL) score: 78.125

Psychosocial burden (Psy-SoB) score: 83.33333333333333333333

Therapeutic relationship (TR) score:41.66666666666666666667

Total MRB-QoL Score: 73.3870967741935483871

Please review these scores as they may be relevant for clinical medication review.

Best regards,

SQA

Recipient Data:

Time Finished: 2024-10-02 12:01:39 +04

IP: 37.245.197.44

ResponseID: R_4AKl4O3jSbbu3O6

Link to View Results: Click Here

Response Summary:

يرجى كتابة رقم التعريف الخاص للمريض ، والذي تم تخصيصه من قبل فريق البحث . 12166

١. أجدُ صعوبةً في ترتيبِ أدويتي. ( ٢ ) أوافق

٢. أجدُ صعوبةً في الاحتفاظ بسجلات أدويتي. ( ٢ ) أوافق

٣. من الصعبِ بالنسبةِ لي إدارةُ أموريَ الدوائيّةِ. ( ٢ ) أوافق

٤. أجدُ صعوبةً في إدراجِ أموريَ الدوائيّةِ ضمنَ مهامِّ حياتيَ اليوميةِ الأُخرى.( ٢ ) أوافق

٥. يتعارضُ تناولِ الأدويةِ مع أنشطتيَ اليوميّةِ. ( ٢ ) أوافق

٦. من الصعبِ تحقيقُ التوازنِ بين مهامِّ حياتيَ اليوميةِ مع تناولِ أدويتي.( ٢ ) أوافق

٧. ليس من السهلِ عليَّ متابعةُ نظامِ دوائي بالنسبةِ للجرعاتِ وأنواعِ الأدويةِ (مثل: الحقن والأقرا...( ٢ ) أوافق

٨. إنّ فهمَ التعليماتِ الموجودةِ على أدويتي يمثّلُ تحديًا لي في بعضِ الأحيان .( ٢ ) أوافق

٩. خصائصُ أنواعِ أدويتي الحاليّة ليست مناسبةً لي لتناولها (مثل: صعوبة البلع ، طعم / رائحة كريهة).( ٢ ) أوافق

١٠. أضطرُّ أحيانًا إلى إلغاءِ مهامّيَ اليوميةِ بسبب أدويتي.( ٣ ) محايد

١١. أحيانًا يكون فتحُ عبواتِ أدويتي مهمةً صعبةً عليّ (مثل: الأغطية المقاومة لعبث الأطفال).( ٣ ) محايد

١٢. أشعرُ أحياناً بعدمِ الرِّضا الجنسيِّ بسببِ أدويتي.( ٣ ) محايد

١٣. إنّني غيرُ قادرٍ على الشعورِ بالراحة والاستمتاع الجنسي بسببِ أدويتي.( ٣ ) محايد

١٤. بعضُ الأدويةِ تسبّبُ تراجعاً في صحتيَ الجسدية. ( ٢ ) أوافق

١٥. غالبًا ما يكونُ نومي سيئًا بسببِ الأدويةِ التي أتناولها.( ١ ) أوافق بشدة

١٦. تسبّبُ لي أدويتيَ التعبَ أثناءَ ممارسةِ الأنشطةِ البدنية.( ١ ) أوافق بشدة

١٧. تؤثرُ أدويتي سلبًا على مستوى أدائي في العملِ أو إنجازِ مهاميَ اليومية.( ٢ ) أوافق

١٨. أشعرُ بعدمِ الارتياحِ الناتجِ عن الآثار الجانبية لبعضِ الأدويةِ التي أتناولها. ( ١ ) أوافق بشدة

١٩. أدويتي تشيرُ إلى أنّني لستُ بصحةٍ جيدةٍ.( ٢ ) أوافق

٢٠. يزعجني أنّني يجبُ أن أتناولَ الأدويةَ على المدى الطويل.( ١ ) أوافق بشدة

٢١. أنا قلِقٌ بشأن عددِ الأدويةِ التي أتناولُها.( ١ ) أوافق بشدة

٢٢. إنّني قلِقٌ بشأنِ الآثارِ الطويلةِ المدى للأدويةِ على صحتي.( ١ ) أوافق بشدة

٢٣. تناولُ الأدوية بانتظامٍ يذكّرني بمشاكليَ الصحيةِ.( ٢ ) أوافق

٢٤. أنا قلِقٌ من إمكانيةِ تفاعلِ أدويتي مع بعضها البعض. ( ٢ ) أوافق

٢٥. أُفَضِّلُ عدمَ إخبارِ الآخرينَ أنني أتناولُ الأدوية بانتظامٍ.( ٢ ) أوافق

٢٦. أشعرُ بالحَرَجِ عند استخدام أدويتي في الأماكن العامة.( ٢ ) أوافق

٢٧. أشعرُ بالوصمةِ (التمييز والتصنيف) بسبب ما يقولُه الناسُ عن الأدويةِ التي أتناولها.( ٢ ) أوافق

٢٨. إذا اكتشفَ الناسُ أنّني أتناول الأدوية، فسوف يرَونَني ضعيفًا. ( ٢ ) أوافق

٢٩. أنا لا أُعامَلُ باحترامٍ و تقديرٍ كمريض.( ٣ ) محايد

٣٠. لا يُراعي طبيبي صحتيَ الجسدية و العقلية والروحية.( ٣ ) محايد

٣١. لا يتم إشراكي فيما يخصُّ أدويتي من قبل أطبائي.( ٤ ) لا أوافق

**Table S3**

Drug-related problems: Types, causes, and clinical pharmacist recommendations

| Domain | Subdomain | | N(%) |
| --- | --- | --- | --- |
| Drug-related problems | Treatment effectiveness | Therapy failure | 11 (4.8%) |
|  |  | Suboptimal drug effect | 31 (13.7%) |
|  |  | Wrong effect of the drug | 5 (2.2%) |
|  |  | Untreated indication | 65 (28.6%) |
|  |  | NA | 115 (50.7%) |
|  | Adverse Reactions | ADE (non allergic) | 75 (33%) |
|  |  | ADE (allergic) | 2 (0.9%) |
|  |  | NA | 146 (64.3%) |
|  | Treatment Cost | expensive therapy | 40 (17.6%) |
|  |  | unnecessary drugs | 33 (14.5%) |
|  |  | NA | 154 (67.8%) |
|  | Other | Unclear problem | 40 (17.6%) |
|  |  | Medication-related burden and Low Quality of life | 60 (26.4%) |
|  |  | NA | 127 (55.9%) |
| Causes of drug-related problems | Drug Selection | inappropriate drug | 32 (14.1%) |
|  |  | No indication of drug | 18 (7.9%) |
|  |  | Wrong combination of drug | 5 (2.2%) |
|  |  | Drug duplication | 4 (1.8%) |
|  |  | Multiple drugs | 41 (18.1%) |
|  |  | Cost-effective drug | 14 (6.2%) |
|  |  | NA | 113 (49.8%) |
|  | Dosing Error | inappropriate drug form | 9 (4.4%) |
|  |  | low dose | 5 (2.2%) |
|  |  | wrong frequency | 1 (0.4%) |
|  |  | No monitoring | 43 (18.9%) |
|  |  | P. Kinetics dose adjustment | 1 (0.04%) |
|  |  | Disease dose adjustment | 10 (4.4%) |
|  |  | NA | 158 (69.6%) |
|  | Drug use process | inappropriate dosing interval | 8 (3.5%) |
|  |  | drug underused | 5 (2.2%) |
|  |  | drug overused | 12 (5.3%) |
|  |  | The patient unable to take the prescribed dosage form | 10 (4.4%) |
|  |  | wrong drug taken | 11 (4.8%) |
|  |  | NA | 181 (5.7) |
|  | Logistics | prescribed drug not available | 1 (0.4%) |
|  |  | prescribing error (necessary information missing) | 26 (11.5%) |
|  |  | NA | 200 (88.1%) |
| Clinical Pharmacist Recommendation | At prescriber level | informed only | 48 (21.1%) |
|  |  | asked for information | 69(30.4%) |
|  |  | approved by prescriber | 28 (12.3%) |
|  |  | not approved | 1 (0.4%) |
|  |  | NA | 68(29.9%) |
|  |  | outcome known | 13 (5.7%) |
|  | At patient level | patient counselling | 112 (49.3%) |
|  |  | written information provided. | 23 (10.1%) |
|  |  | referred to prescriber | 49 (21.6%) |
|  |  | spoken to family member | 4 (1.85) |
|  |  | NA | 39 (17.6%) |
|  | At drug level | drug change | 20 (8.8%) |
|  |  | dosage change | 3 (1.3%) |
|  |  | formulation change | 8 (3.5%) |
|  |  | instruction of use | 31 (13.7%) |
|  |  | drug stopped | 15 (6.6%) |
|  |  | new drug started | 54 (23.8%) |
|  |  | others | 11 (4.8%) |
|  |  | NA | 85 (37.4%) |
|  | Others | Other intervention | 53 (23.3%) |
|  |  | Side effects reported to authorities | 10 (4.4%) |
|  |  | NA | 164 (72.2%) |

**Fig S1**

**Stem-and-leaf plot highlighting theme-based code usage across research questions**

**Table S4**

Themes, codes, and quotations: How MRB-QoL scores support clinical pharmacists in patient medicines optimisation

| Domain | Theme | Description | Code | Related Quotes |
| --- | --- | --- | --- | --- |
| **RRC** | Regimen simplification | Streamlining medicines regimens to reduce complexity and dosage frequency. | Combine medicines | Medication dose, form, frequency, and use are OK, but maybe I need to combine three antibiotics in one drug and provide a pill organiser.(CP1) |
|  |  |  | Reduce medicines frequency | Combine H pylori medications to reduce the frequency of medications.(CP5) |
|  |  |  | Simplify regimen | Simplify the medication regimen by using pill organisers or blister packs. Adjust the regimen to reduce complexity.(CP9) |
|  |  |  | Suggest once-daily formulations | Nexium can be taken once daily of higher strength to reduce complexity. Simplify the regimen by taking once daily sustained release formulation.(CP5) |
|  |  |  |  |  |
|  | Adherence support | Providing tools and strategies to support adherence, such as reminders, pill organisers, and timing adjustments. | Use of pill organiser | Advise the patient to use a pill organiser. Keep pill organiser for visual instruction tools.(CP2) |
|  |  |  | Alarm and apps as reminders | Alarm and some applications can help as a reminder. Use reminder software to help with short-term medications.(CP3) |
|  |  |  | Align administration times | Align administration time for all medications if possible. Synchronise administration times to reduce patient’s daily routine complexity.(CP1) |
|  |  |  | Medicines timing education | Educate on time management techniques to balance medications with daily responsibilities.(CP9) Explain consequences of skipping doses and advise reminders.(CP4) |
|  | Clear usage instructions | Ensuring patients understand how to properly take their medicines with clear, tailored instructions | Clear instructions and demonstrations | Provide clear, written instructions and demonstrations for proper medication use. Ensure instructions are clear and offer language support if needed.(CP7) |
|  |  |  | Create written schedule | Patients may require a written schedule to help with DM meds. Provide a written schedule to detail the dosage timing.(CP6 |
|  |  |  |  |  |
|  |  |  |  |  |
|  | Medicines necessity assessment | Evaluating the need for each medicines to avoid unnecessary treatments and manage polypharmacy effectively. | Medicines reconciliation needed | Medication reconciliation is needed to review all the medications and omit unnecessary ones. (CP9) Review treatment as the patient may have medications from other hospitals. (CP1) |
|  |  |  | Assess medicines appropriateness | All medications should be evaluated for appropriateness. Revise the need for PRN medications like loratadine and paracetamol.(CP10) |
|  |  |  | Review PRN necessity | There is no indication for paracetamol with caffeine and antihistamine; review PRN medication necessity.(CP5) |
|  |  |  |  |  |
|  | Dosage form adjustments | Modifying medicines forms to meet patient needs, especially for easier administration or fewer side effects. | Alternative dosage forms | Consider alternative dosage forms that are easier to administer.(CP3) |
|  |  |  |  |  |
|  |  |  | Address swallowing issues | Highlighted possible problems with swallowing/taking dosage forms. Switch to forms that are easier to take.(CP4) |
|  |  |  |  |  |
|  | Lifestyle integration support | Educating patients to integrate medicines routines into their daily lives, including time management and lifestyle adaptations. | Time management education | Educate on time management techniques to balance medications with daily responsibilities.(CP10) Provide time management strategies.(CP7) |
|  |  |  | Incorporate medicines into routine | Offer strategies to incorporate medication into daily routines, such as setting alarms.(CP1) Incorporate medication into daily routine for patient adherence.(CP3) |
|  |  |  | Counsel on diet and lifestyle | Counsel on diet and lifestyle to control constipation and GERD. Provide lifestyle and dietary counseling to support medication adherence.(CP10) |
|  |  |  | Support routine integration | Encourage the patient to use reminder applications and pill organisers for regimen support.(CP5) Assist patient in setting routines to support medication intake.(CP2) |
| **FRL** | Side effect management | Identifying and reducing medicines side effects to improve patient function and quality of life. | Consider safer medicines | Consider medications that do not cause drowsiness or fatigue.(CP2) |
|  |  |  | Manage side effects | Atorvastatin can cause myalgia; consider switching to Rosuvastatin if possible.(CP4) For patients suffering from side effects, I have to find medicine with fewer side effects.(CP8) |
|  |  |  | Mitigate impact on daily living | Explore options to mitigate side effects that affect productivity and daily living. Provide remedies for common side effects and reassess medication choices.(CP3) |
|  |  |  | Medicines necessity assessment | Could re-evaluate the use of beta blockers and consider ACE/ARB if blood pressure control is adequate.(CP9) |
|  | Condition and therapy review | Regular evaluation of untreated conditions, therapy adjustments, and the need for alternative treatments. | Managing untreated conditions | There are untreated conditions (rheumatoid arthritis and H. pylori) that may be received from other hospitals, and all medications should be checked.(CP7) |
|  |  |  | Functional disability from side effects | This could be due to untreated IBS, side effects of beta-blockers, or a drop in blood pressure if medications are grouped together.(CP9) |
|  |  |  | Condition reassessment | The present conditions need to be assessed if properly treated (H. pylori vs prevention of recurrence).(CP5) |
|  | Medicines and health education | Educating patients on medicines' importance and benefits and monitoring adherence to therapy. | Patient education on health benefits | Educate the patient on the importance of medication in managing health and improving quality of life.(CP7) |
|  |  |  | Education on medicines importance | I educated the patient on the importance of taking the medications.(CP2) |
|  |  |  | Medicines timing and adjustment | Evaluate and possibly adjust the timing or type of medication.(CP10) |
|  | Specialist referral and support | Referral for complex cases or when specialised intervention is needed to support functional limitations. | Referral to specialist | I referred the patient to a specialist.(CP4) |
|  |  |  | Physical therapy recommendation | Low back pain, if it is a chronic condition and fastum gel is not enough, try physical therapy.(CP8) |
|  |  |  | Close monitoring for side effects | Prucalopride can cause dizziness, diarrhea, suicidal behavior; counsel family for close observation.(CP1) |
|  | Functional improvement support | Supporting daily functioning by addressing medicines impacts on physical abilities and lifestyle. | Nutritional supplementation | Nutritional anemia can cause fatigue; ensure suitable supplementation (vitamin B12, folic acid, iron, etc.).(CP7) |
|  |  |  | Manage functional limitations | IBS symptoms causing functional limitation; need better management and counseling.(CP3) |
|  | Lifestyle and physical health monitoring | Monitoring physical health, side effects, and lifestyle integration for better quality of life. | Health monitoring and adjustment | Regularly monitor physical health and adjust medications as necessary.(CP1) |
|  |  |  | Control chronic disease | Uncontrolled diabetes can exacerbate neuropathy and fatigue; counsel the patient for proper control.(CP5) |
|  |  |  | Symptomatic care for chronic disease | Optimise care for chronic disease patients to manage symptomatic care and improve daily living.(CP3) |
|  |  |  | Counsel on lifestyle modification | Counsel for lifestyle modification and appropriate diet to manage gastritis symptoms.(CP10) |
| **PsySB** | Counselling and support | Offering counselling, reassurance, and support to address psychosocial burden. | Education on monitoring | Reassure and educate on the monitoring processes to prevent interactions.(CP8) |
|  |  |  | Long-term therapy support | Offer support and counselling to address feelings about long-term therapy.(CP1) |
|  |  |  | Social and emotional support | Social and emotional support is needed to address the patient's feelings about being on long-term medications. |
|  |  |  | Reassurance of medicines importance | I offered support for the patient to address their feelings and assure the importance of taking medications.(CP8) |
|  |  |  | Counselling to address patient feelings | It helped me to offer counselling to address the feelings of the patient.(CP3) |
|  | Health education | Educating patients on medicines importance, risks, and managing health. | Health benefits education | Educate the patient on the importance of medication in managing health and improving quality of life.(CP10) |
|  |  |  | Health literacy awareness | As it is relatively low, it reflects that the patient has health literacy about the condition.(CP1) |
|  |  |  | Addressing fear and treatment clarity | The patient feels that her disease is not controlled; Metformin alone could be insufficient, so check for add-on medications.(CP9) |
|  |  |  | Information on risks vs. benefits of long-term medicines | Provide information on the benefits versus risks of long-term medication use.(CP1) |
|  | Polypharmacy management | Conducting medicines reviews to address polypharmacy and reduce burden. | Deprescribing | Conduct a medication review to reduce polypharmacy where possible. The score is high; reduce the number of medications to decrease cost and ease of use.(CP8) |
|  |  |  | Medicines review and monitoring | The high score reflects the need for a proper and thorough medication review to check for side effects and ensure monitoring.(CP10) |
|  | Addressing stigma | Providing support for issues like stigma and discrimination associated with medicines use. | Address discrimination and judgment | Offer counselling to address feelings of discrimination or judgment.(CP2) |
|  |  |  | Open dialogue to reduce stigma | Promote an open dialogue and provide support to reduce stigma.(CP2) |
|  | Chronic condition support | Support for managing chronic conditions and associated psychological impacts. | Impact of chronic conditions on mental health | The fact that the patient is suffering from a neurogenic bladder causes a huge social burden.(CP4) |
|  |  |  | Treatment for untreated conditions | Patient not treated for disease; requires referral for assessment by doctor.(CP7) |
|  |  |  | Referral for clinical review | The patient must be referred to a clinician to review conditions and therapy.(CP9) |
|  | Family and community engagement | Engaging family, community, and support groups for enhanced psychosocial support. | Family support | Family support and positive encouragement regarding adherence to medication therapy are essential.(CP5) |
|  |  |  | Social and medical support | Need support from society and medical staff.(CP2) |
|  |  |  | Engage family support | Engage family and support group for long-term management.(CP5) |
| **TR** | Building trust and empathy | Establishing trust by addressing gaps and misunderstandings. | Highlight treatment conflict | Patient feels like medical providers don't care, which is something that has to be addressed and corrected.(CP8) |
|  |  |  | Address dissatisfaction through discussion | The patient had dissatisfaction with healthcare providers. Encourage discussion between the team and the patient and enhance communication.(CP3) |
|  |  |  | Encourage addressing concerns | Maintain open communication; encourage the patient to address concerns or get back to him with any questions.(CP9) |
|  |  |  | The shared decision reduces fear | Shared decision-making will decrease the patient's fear.(CP2) |
|  |  |  | Assess relationship quality | Gave indication that patient has a good relationship with providers.(CP8) |
|  |  |  | Bridge information gap | Patient interviews will be important to understand the gap in the relationship.(CP6) |
|  |  |  | Promote trust building | This signifies that the patient thinks health providers don't care, and needs a patient interview to fix wrong perceptions.(CP4) |
|  |  |  | Encourage continuous feedback | She is pretty satisfied with her healthcare provider; encourage open discussion and continuous feedback.(CP9) |
|  | Holistic health approach | Addressing all aspects of patient health, including mental and emotional needs. | Patient-centred communication | Patient-centred care with proper communication.(CP1) |
|  |  |  | Physical and mental consideration | The patient is satisfied and considered physically, mentally, and spiritually.(CP5) |
|  |  |  | Correlation with true outcome | Helped me in correlating this value with the patient’s true outcome.(CP1) |
|  |  |  | Holistic care approach | Promote holistic care and address all aspects of health.(CP7) |
|  |  |  | Address all health aspects | Encourage addressing all aspects of health.(CP6) |
|  | Patient-centred communication | Fostering clear, empathetic, and supportive communication. | Promote patient-centred care | Patient-centred care with proper communication.(CP4) |
|  |  |  | Explain chronic condition care | Chronic conditions like diabetes and dyslipidemia need explanation, lifestyle modification, and a lot of patient engagement in the treatment plan.(CP7) |
|  |  |  | Improve communication | Patient-centred care with improved communication is needed.(CP7) |
|  |  |  | Encourage patient engagement | Patient engagement is encouraged.(CP9)  Involve the patient in decision-making and consider his fears and issues, while keeping all his information private.(CP8) |
|  |  |  | Respect patient privacy | Need patient interview and refer to diabetes to manage IBS and provide support.(CP1) |
|  |  |  | Provider-patient relationship | Gave indication that patient has a good relationship with providers.(CP4) |
|  |  |  | Understanding relationship gaps | Patient interviews will be important to understand the gap in the relationship. Does the patient have untreated conditions?(CP10) |
|  |  |  | Foster open discussion | Encourage open discussion and patient-centered care.(CP5) |
|  | Positive health reinforcement | Encouraging adherence and positive health attitudes. | Encourage medicines adherence | Taking all into consideration, it appears that the patient thinks medical providers are here to help him.(CP7) |
|  |  |  | Positive reinforcement for health | Encourage positive reinforcement and focus on the benefits of maintaining health through medication.(CP4) |
|  | Provider engagement support | Ensuring patient support through regular provider engagement. | Regular provider meetings | Meet healthcare providers regularly.(CP6) |
|  |  |  | Explain medicines purpose | Explain the purpose of medication and its components.(CP8) |
|  |  |  | Improve medicines adherence | Improve medication adherence by addressing patient’s challenges with timing.(CP6) |
|  |  |  | Medical staff support | The patient needs to meet with the doctor, and the doctor should listen carefully to provide the best treatment.(CP7) |
|  |  |  | Supportive information provided | Provide supportive information about his health and needs.(CP10) |
|  | Shared decision-making | Engaging patients in decision-making for personalised care. | Shared decision-making | Encourage shared decision-making and involve patients in their treatment plans.(CP9) |
|  |  |  | Shared decision for chronic care | His chronic conditions necessitate shared decisions with the patient.(CP1) |
|  |  |  | Encourage patient engagement | The patient should be engaged with his treatment plan, as his awareness of his conditions and complications will enhance adherence.(CP3) |
|  |  |  | Patient-centred care and communication | Patient-centered care with proper communication.(CP10) |
|  |  |  | Patient involvement in treatment | The patient needs to be involved in the treatment plan and to be shown more empathy and respect.(CP9) |
|  |  |  | Promote open communication | Open communication, shared decision.(CP1) |
| **MRB-QoL** | Managing side effects | Identifying and managing side effects to improve adherence and health. | Identifying side effects | Help in identifying side effects of some medications which change dosage form leads to improved adherence and enhanced quality of life.(CP3) |
|  |  |  | Reduction in side effects | Reduction in side effects.(CP4) |
|  |  |  | Medicines addition recommendations | Recommended adding new medication for Hypercholesterolemia.(CP7) |
|  |  |  | Dose formulation adjustment | Change dose formulation and improve adherence.(CP9) |
|  | Enhancing adherence | Enhancing adherence through patient counselling, monitoring, and support. | Understanding patient challenges | Helped to better understand the challenges patients face with their treatment plans and more patient-centered care.(CP4) |
|  |  |  | Improving adherence | Improving adherence, ultimately enhancing quality of life.(CP3) |
|  |  |  | Patient counselling | Patient counselling.(CP1) |
|  |  |  | Clear instruction | Increase adherence by clear instruction and recommended tonic for iron deficiency anemia.(CP5) |
|  |  |  | Dose formulation adjustment | Change dose formulation and improve adherence.(CP2) |
|  | Improving quality of life | Improving patient quality of life through reduced burden and symptom management. | Impact on quality of life | Medication-related burden affects the quality of life.(CP3) |
|  |  |  | Plan to reduce the burden | Plan to reduce each medication-related burden.(CP10) |
|  |  |  | Lifestyle advice and monitor | Advice on lifestyle modifications, add fibers, monitor efficacy of prucaloride.(CP3) |
|  |  |  | Focus on symptom management | Review all medications and focus on symptom management.(CP2) |
|  | Patient-centred communication | Providing patient-centred care and communication for better understanding and engagement. | Clarification of treatment purpose | Patient interviews will help clarify treatment outcomes and address missing information.(CP4) |
|  |  |  | Patient Education | The patient needs help in education, reducing functional limitations, and supporting full adherence.(CP10) |
|  |  |  | Encourage open communication | Encourage addressing concerns or questions.(CP4) |
|  |  |  | Discuss medicines burden openly | Patients should be involved in decision-making about their treatment, especially medication burden.(CP2) |
|  | Deprescribing | Medication reviews and reconciliation to address polypharmacy and interactions. | Medicines reconciliation needed | Medication reconciliation, reviews PRN medication and check treatments from other facilities.(CP9) |
|  |  |  | Reduce drug-drug interaction | Reduce drug-drug interaction by educating and counseling.(CP5) |
|  |  |  | Medication reviews and adjustment | High score, change medication plan to reduce daily doses and side effects.(CP7) |
|  |  |  | Reassess therapy needs | Reassess the need for IBS medication.(CP1) |
|  | Chronic condition support | Supporting chronic disease management and reducing long-term complications. | Control complications | Priority to reduce diabetes limitations for this patient.(CP5) |
|  |  |  | Manage untreated conditions | Untreated conditions highlight the need for prescriber support.(CP4) |
|  |  |  | Reduce symptoms | The primary goal is to reduce IBS symptoms.(CP2) |
|  |  |  | Enhance neurological pain management | Highlighted the need for better management of neuralgia and support for overall pain.(CP1) |

MRB-Qol: Medication-related burden quality of life, RRC: Routine and regimen complexity, PsyB: Psychological burden, FRL: Functional and role limitation, TR: Therapeutic relationship, SB: Social burden, PsySB: Psychosocial burden, CP: Clinical pharmacist, PRN: as needed, IBS: Irritable bowel syndrome.
